# Biofortification of Sodium Selenate Improves Dietary Mineral Contents and Antioxidant Capacity of Culinary Herb Microgreens

**DOI:** 10.3389/fpls.2021.716437

**Published:** 2021-08-05

**Authors:** Rachel G. Newman, Youyoun Moon, Carl E. Sams, Janet C. Tou, Nicole L. Waterland

**Affiliations:** ^1^Division of Animal and Nutritional Sciences, West Virginia University, Morgantown, WV, United States; ^2^Division of Plant and Soil Sciences, West Virginia University, Morgantown, WV, United States; ^3^Department of Plant Sciences, The University of Tennessee, Knoxville, TN, United States

**Keywords:** selenium, biofortification, antioxidants, minerals, herbs, microgreens

## Abstract

Selenium biofortification of plants has been suggested as a method of enhancing dietary selenium intake to prevent deficiency and chronic disease in humans, while avoiding toxic levels of intake. Popular herbs such as basil (*Ocimum basilicum* L.), cilantro (*Coriandrum sativum* L.), and scallions (*Allium fistulosum* L.) present an opportunity for biofortification as these plants are used for added flavors to meals and are available as microgreens, young plants with increasing popularity in the consumer marketplace. In this study, basil, cilantro, and scallion microgreens were biofortified with sodium selenate under hydroponic conditions at various selenium concentrations to investigate the effects on yield, selenium content, other mineral contents (i.e., sodium, potassium, calcium, magnesium, phosphorus, copper, zinc, iron, manganese, sulfur, and boron), total phenol content, and antioxidant capacity [oxygen radical absorbance capacity (ORAC)]. The results showed that the selenium content increased significantly at all concentrations, with scallions demonstrating the largest increase. The effects on other minerals varied among herb species. Antioxidant capacity and total phenol content increased in all herbs at the highest selenium treatments, but basil and scallions demonstrated a decreased crop yield. Overall, these biofortified culinary herb microgreens are an ideal functional food for enhancing selenium, other dietary minerals, and antioxidants to benefit human health.

## Introduction

Selenium (Se) is an essential trace mineral in humans and a component of biologically important selenoproteins, such as antioxidant enzymes (Navarro-Alarcon and Cabrera-Vique, 2008). While Se deficiency is uncommon in North America, it is estimated that one in seven people worldwide have inadequate intake of dietary Se, and the prevalence of Se deficiency may increase with climate change (Jones et al., 2017). Supplementation of Se has been suggested for the prevention of chronic disease; however, high-dose supplementation can increase the risk of Se toxicity (Navarro-Alarcon and Cabrera-Vique, 2008). Plants are a major dietary source of Se, and biofortification is a method of enhancing the Se content in plants to improve adequacy of the human diet (Gupta and Gupta, 2017). Biofortification with selenate demonstrates maximum Se translocation to the edible shoots of plants, whereas selenite accumulates in the roots (Terry et al., 2000; Saha et al., 2017). The use of hydroponic growing conditions further allows for highly controlled Se application (Puccinelli et al., 2017a), making the addition of sodium selenate (Na_2_SeO_4_) to nutrient solutions an efficient biofortification method.

Different plant species exhibit varying levels of Se tolerance rather than essentiality (Terry et al., 2000). The goal of Se biofortification is to increase the Se content while avoiding significant decreases in the crop yield (Puccinelli et al., 2017a). While this suggests a trade-off between supplying supra-adequate levels of Se to the diet and detrimental effects on plant growth, Navarro-Alarcon and Cabrera-Vique (Navarro-Alarcon and Cabrera-Vique, 2008) noted that plants serve as an effective buffer to Se toxicity in humans because of a decreased yield at high levels. Studies have also reported that Se biofortification can impact other dietary minerals and antioxidant compounds, such as polyphenols, which have anticancer and cardioprotective properties (He et al., 2004; Hawrylak-Nowak, 2008; Boldrin et al., 2013; Saffaryazdi et al., 2012). Therefore, it is imperative that studies investigating Se biofortification include other nutrients that are relevant to human health.

Culinary herbs are added to meals for flavor to replace salt and can increase consumer acceptance of vegetables (Manero et al., 2017). Popular culinary herbs, such as basil (*Ocimum basilicum* L.), cilantro (*Coriandrum sativum* L.), and scallions (*Allium fistulosum* L.), present an opportunity for Se biofortification as small, flavorful additions to meals. Furthermore, microgreens are increasingly popular among consumers for their distinct flavors (Choe et al., 2018). Microgreens are young plants typically consisting of the stem, cotyledons, and a pair of true leaves (Choe et al., 2018), making microgreen herbs a quick and easy crop to biofortify. Few studies have investigated Se biofortification of herb microgreens. The objective of this study was to investigate the effect of Se biofortification on plant yield, total Se content, content of other minerals relevant to human health, and antioxidants in three culinary herb species grown to microgreen stage under hydroponic conditions. This study tests the hypothesis that biofortification of culinary herb microgreens increases the content of Se, other minerals important to human health, and antioxidants. Enhancements in nutrient profiles would enable these herbs to serve as a functional food for improving human nutrition.

## Materials and Methods

### Plant Growth and Harvest

Seeds of basil, cilantro, and scallions were purchased from Johnny's Selected Seeds (Winslow, ME). The seeds were sown in 25.4 × 25.4 × 2.54 cm black growing trays onto Biostrate felt mats (0.35 cm thickness; Grow-Tech, South Portland, ME). The seed density per tray for each species was as follows: 2.8 seeds per cm^2^ of basil seeds, 2.8 seeds per cm^2^ of cilantro seeds, and 3.4 seeds per cm^2^ of scallion seeds. Treatments for all species consisted of 0.5 × concentrated Hoagland's modified nutrient solution (PhytoTechnology Labs, Shawnee Mission, KS) prepared in ultrapure water (18.2 MΩ·cm) and supplemented with Na_2_SeO_4_ (VWR, Radnor, PA) concentrations of 0.0 (control), 2.5, and 5.0 mg/L of Se. Scallions received an additional treatment with 10.0 mg/L of Se as a member of *Allium* plant family which is known for the ability to accumulate Se levels up to 1,000 μg/g dry weight (DW) (Navarro-Alarcon and Cabrera-Vique, 2008).

The treatments were completed with three replicates, where each tray represented a replicate, and mineral contents, total phenolic content (TPC), and oxygen radical absorbance capacity (ORAC) were measured for each replicate. Based on preliminary studies with low yields, scallions were grown in two trays per replicate, which were combined to obtain enough sample for various analyses. The plant yield was analyzed as grams per tray. Each tray received an initial 180 ml of a 0.5 × concentrated Hoagland's nutrient solution. The trays were placed into growth chambers (Percival Scientific, Perry, IA) with a photoperiod of 16/8 h light/dark, temperature of 19.7/18.7 ± 1.0/0.8°C day/night, relative humidity of 57.3 ± 0.8%, and photosynthetic photon flux density of 210 ± 7.9 μmol/m^2^/s (mean ± standard deviation [SD]). The average value of pH of all nutrient solutions was 5.4 ± 0.1. The plants were sprayed with plain ultrapure water as needed to avoid drying of seeds during germination. Treatment began when 80% of seeds were germinated for each species. The plants were fertigated as needed by adding solutions directly to the growing mats based on the mat saturation within plant species. Each tray within plant species received the same volume of treatment every day. The total volume of treatment per tray for each species over the 25-day treatment period was 2,965, 2,835, and 3,825 mL for basil, scallions, and cilantro, respectively.

Each plant species was harvested on the 25th day of treatment, at which point basil and cilantro had two true leaves and scallions had one true leaf. Microgreens were harvested at the base, approximately 1.0 cm from the growing pad. The seed coats were removed as needed, and fresh weight (FW) per tray was recorded. Microgreens were immediately flash frozen with liquid nitrogen, and stored at −80°C until freeze-dried (Virtus Genesis 35 SQ Super XL, SP Industries, Gardiner, NY). Dried samples were weighed, ground to a fine powder, and stored at −80°C until analyses. The water content of microgreens was determined for each tray by comparing DW with FW. All measured parameters were converted to FW basis, as microgreens are consumed exclusively fresh.

### Mineral Analysis

The measurement of minerals was completed according to Barickman et al. (2012). In brief, 0.5 g of lyophilized ground plant tissue was mixed with 10 ml of 70% HNO_3_ and was digested in a microwave digestion system (Ethos model, Milestone Inc., Shelton, CT, USA). Digestion procedures for organically based matrixes were followed U.S. Environmental Protection Agency (1996). The digested samples were diluted with 2% HNO_3_/0.5% HCl (v/v). The samples were analyzed for Se, sulfur (S), sodium (Na), potassium (K), phosphorus (P), calcium (Ca), magnesium (Mg), copper (Cu), iron (Fe), manganese (Mn), zinc (Zn), and boron (B) by inductively coupled plasma mass spectrometry (ICP-MS) using an Agilent 7500ce ICP-MS system (Agilent Technologies, Inc., Wilmington, DE, USA) equipped with an ASX-510 (CETAC, Omaha, NE, USA) autosampler.

### Determination of Total Phenolic Content

Total phenolics were extracted using the method of Nicolle et al. (2004) with some modifications. In brief, 10 mg of lyophilized ground plant tissue was extracted using 1 ml of methanol–water mixture (60:40, v/v), vortexed for 10 s, and centrifuged at 16,110*g* for 20 min at 4°C. The supernatant was collected, and the extraction process was repeated. Supernatants were combined and stored at −80°C until analysis. TPC was determined with modifications for a 96-well microplate according to Waterland et al. (2019). In brief, 18.2 μL of sample extract or gallic acid standard was plated in duplicates, followed by 90.9 μL of 0.5 N Folin–Ciocalteau reagent (Sigma-Aldrich, St. Louis, MO). The plate was incubated for 5 min, and then 90.9 μL of 0.5 M sodium carbonate was added to each well. The plate was incubated at room temperature in the dark for 1 h. The absorbance was measured at 765 nm with a BioTek Synergy H1 (Winooski, VT) microplate reader. The interassay coefficient of variation was 5.7%. TPC was determined using a gallic acid standard curve and expressed in mg of gallic acid equivalents (GAE) per gram of FW.

### Oxygen Radical Absorbance Capacity Assay

Sample preparation for hydrophilic ORAC assay was completed according to Ou et al. (2013). In brief, 10 mg of lyophilized ground plant tissue was added to a microcentrifuge tube with 0.4 mL of acetone–water mixture (50:50, v/v) and vortexed at room temperature for 1 h. The tube was then centrifuged at 16,110*g* for 15 min at 4°C. The supernatant was collected and stored at −20°C until assayed. The sample preparation for the lipophilic assay was completed according to OxiSelect ORAC Activity Assay kit protocol (CellBio Labs, San Diego, CA). In brief, 10 mg of lyophilized ground plant tissue was added to a microcentrifuge tube with 0.4 mL of pure acetone and vortexed at room temperature for 1 h. The tube was stored at −20°C until assayed, and the supernatant was separated from the tissue. The ORAC assays were completed by following the OxiSelect ORAC Activity Assay kit protocol (CellBio Labs, San Diego, CA). Fluorescence was read using a Biotek Synergy H1 (Winooski, VT) microplate reader. The interassay coefficient of variation was 21.0%. Results were expressed in μmol Trolox equivalents per gram of FW. The lipophilic and hydrophilic portions were summed for measuring the total ORAC values.

### Statistical Analysis

Due to scallions having an additional treatment group, one-way ANOVA was used to determine the differences among treatment groups for each herb species. *Post hoc* multiple comparison tests were performed using Tukey's test with significance at *P* ≤ 0.05. A two-way ANOVA was used to determine potential interaction effects of plant species and Se treatments (excluding 10.0 mg/L Se) on minerals, TPC, and ORAC. To determine the differences in Se content in response to concentrations of Se treatment among fresh scallions, basil, and cilantro, a regression analysis was combined with ANOVA [analysis of covariance (ANCOVA)]. The analyses were performed using JMP and SAS software (JMP, Version 13, SAS Institute Inc., Cary NC; SAS, Version 9.2, SAS Institute Inc., Cary NC).

## Results

### Selenium Content and Plant Yield

**Figure 1:**
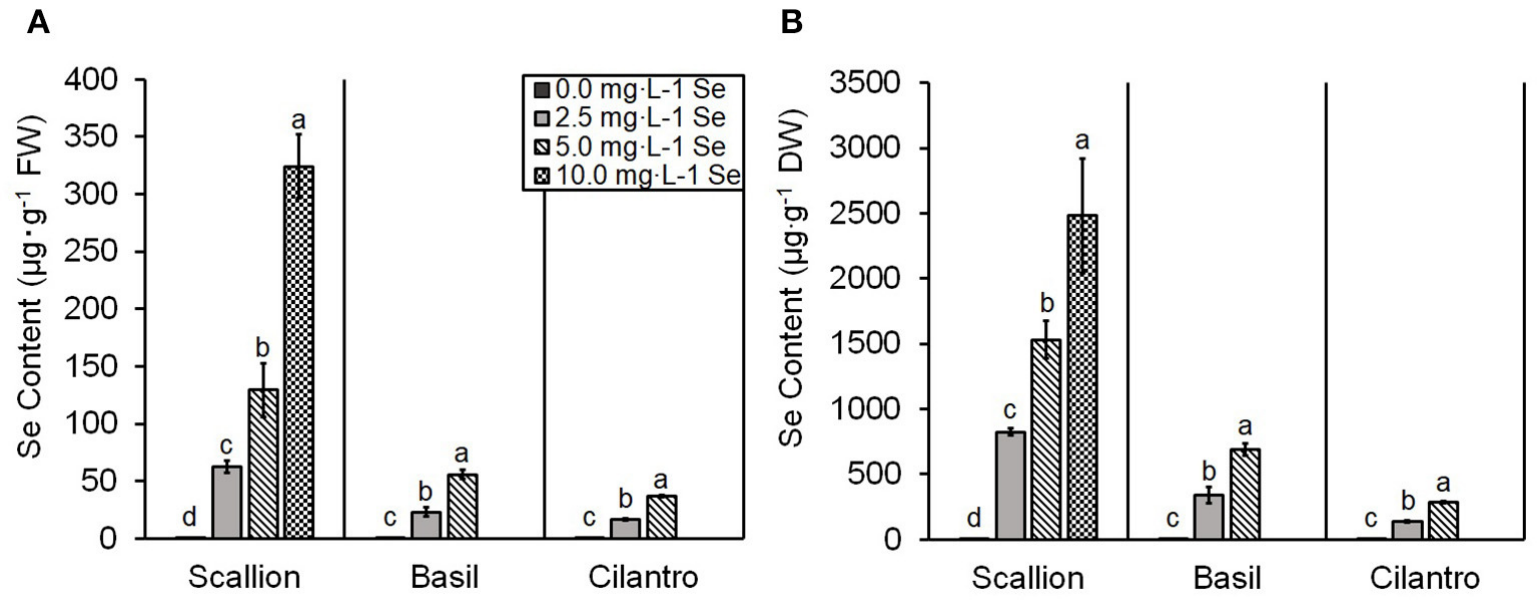
Selenium (Se) content of scallion (*Allium fistulosum* L.), basil (*Ocimum basilicum* L.), and cilantro (*Coriandrum sativum* L.) microgreens treated with various concentrations of Se as sodium selenate, on **(A)** fresh weight (FW) basis and **(B)** dry weight (DW) basis. Bars indicate mean ± standard deviation (SD) for three replicates (*n* = 3) of each plant species. The Tukey's significance at *P* ≤ 0.05 among Se treatments is indicated by different letters within the plant species.

**Figure 2:**
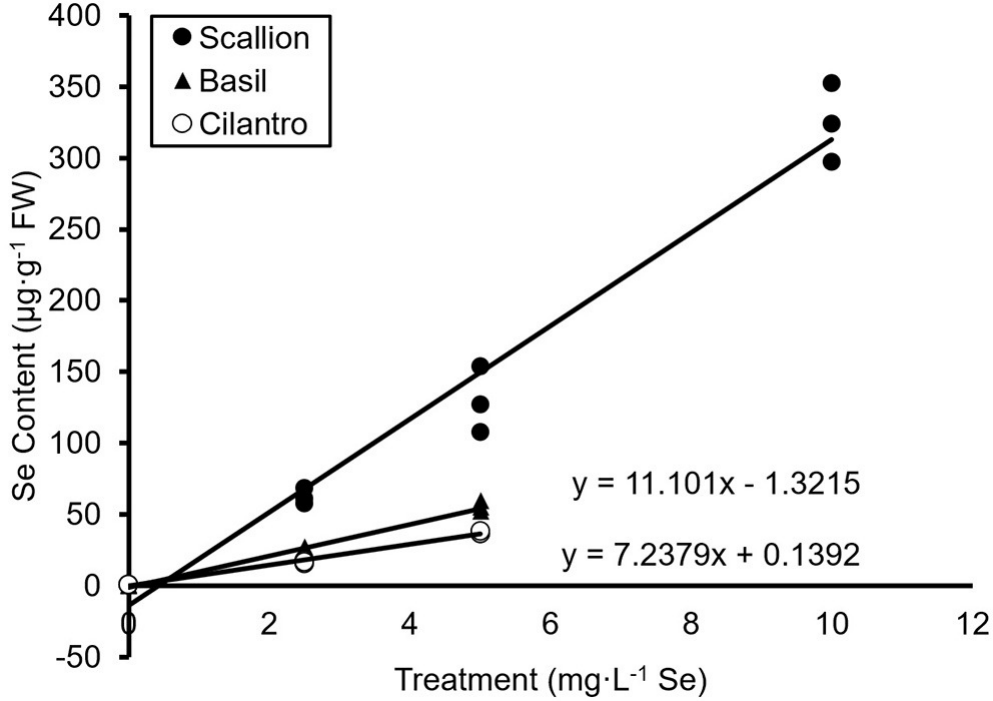
Regression analysis of fresh weight (FW) selenium (Se) content of scallion (*Allium fistulosum* L.), basil (*Ocimum basilicum* L.), and cilantro (*Coriandrum sativum* L.) microgreens treated with various concentrations of Se as sodium selenate (*n* = 3).

The present study showed an interaction of Se treatment and plant species (*P* ≤ 0.0001). Se content increased (*P* ≤ 0.05) with increasing Se treatments for all three culinary herbs (Figure 1). The Se content of fresh scallions increased 98, 202, and 507 times for doses of 2.5, 5.0, and 10.0 mg/L Se, respectively (Figure 1A). The DW values for scallions were 824.9, 1,530.4, and 2,481.4 μg/g Se for 2.5, 5.0, and 10.0 mg/L Se treatments, respectively (Figure 1B). The Se content of fresh basil increased 64 and 155 times at doses of 2.5 and 5.0 mg/L Se (Figure 1A) and accumulated 338.6 and 690.0 μg/g Se DW at 2.5 and 5.0 mg/L Se, respectively (Figure 1B). The Se content of fresh cilantro increased 18 and 40 times at 2.5 and 5.0 mg/L Se treatments (Figure 1A), and cilantro accumulated 136.2 and 287.2 μg/g Se DW at 2.5 and 5.0 mg/L Se, respectively (Figure 1B). ANCOVA showed significant effects (*P* ≤ 0.0001) of Se treatments, plant species, and interaction of Se content and plant species. The regression analysis for concentrations of Se treatment on the Se content were different among plant species (β = 32.63, *P* ≤ 0.0001 for scallions; β = 11.10, *P* = 0.0001 for basil; and β = 7.24, *P* = 0.0058 for cilantro; Figure 2). Fresh scallions demonstrated the highest rate of Se accumulation among the plant species (*P* ≤ 0.0001). The rates of Se accumulation in basil and cilantro did not differ between these two species (*P* = 0.2642).

**Figure 3:**
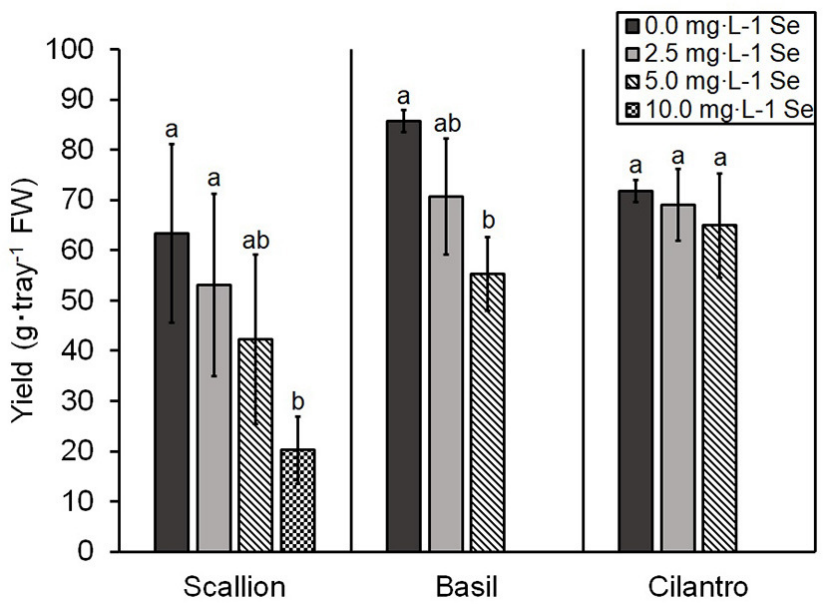
Fresh weight (FW) yield of scallion (*Allium fistulosum* L.), basil (*Ocimum basilicum* L.), and cilantro (*Coriandrum sativum* L.) microgreens treated with various concentrations of selenium (Se) as sodium selenate. Bars indicate mean ± standard deviation (SD) for three replicates (*n* = 3) of basil and cilantro and six replicates (*n* = 6) for scallion. The Tukey's significance at *P* ≤ 0.05 among Se treatments is indicated by different letters within the plant species.

The yield of scallion decreased (i.e., 68.0%) at 10.0 mg/L Se but was not significantly affected at 5.0 mg/L Se (Figure 3). The yield of basil decreased (i.e., 35.5%) at 5.0 mg/L Se (Figure 3). In contrast, the yield of cilantro was not affected by the Se treatments used (Figure 3).

### Other Dietary Minerals

Se biofortification impacted the content of other minerals relevant to human health in fresh herb microgreens. The current study showed an interaction of Se treatment and plant species (*P* ≤ 0.0001) for S content. In cilantro, S content tended to decrease with increasing Se treatments (*P* = 0.1027), while in scallions, S content increased (*P* ≤ 0.05) by 124.5, 140.8, and 228.6% at doses of 2.5, 5.0, and 10.0 mg/L Se, respectively. In basil, S content increased by 46.4 and 96.4% at doses of 2.5 and 5.0 mg/L Se, respectively (Table 1).

**Table 1:** Major mineral content of scallion (*Allium fistulosum* L.), basil (*Ocimum basilicum* L.), and cilantro (*Coriandrum sativum* L.) microgreens treated with various concentrations of selenium as sodium selenate on a fresh weight basis.

**Treatment (mg·L^**−1**^ Se)**	**S**			**Na**			**K**			**P**			**Ca**			**Mg**		
	**SN**	**BL**	**CO**	**SN**	**BL**	**CO**	**SN**	**BL**	**CO**	**SN**	**BL**	**CO**	**SN**	**BL**	**CO**	**SN**	**BL**	**CO**
	**mg·g** ^****−1****^ **FW**																	
0.0	0.49 c	0.28 c	0.18 a	0.003 b	0.005 a	0.009 c	2.25 b	1.62 b	4.71 a	0.38 b	0.33 b	0.61 a	0.97 b	1.03 a	0.90 a	0.33 b	0.30 a	0.49 a
2.5	1.10 b	0.41 b	0.08 a	0.007 b	0.010 a	0.021 b	2.66 b	2.26 ab	4.30 a	0.38 b	0.43 ab	0.59 a	1.13 b	1.24 a	0.79 a	0.39 b	0.38 a	0.44 a
5.0	1.18 b	0.55 a	0.06 a	0.013 b	0.022 a	0.040 a	2.94 ab	2.79 a	4.88 a	0.38 b	0.47 a	0.66 a	1.12 b	1.32 a	0.76 a	0.39 b	0.39 a	0.46 a
10.0	1.61 a	–	–	0.053 a	–	–	4.31 a	–	–	0.56 a	–	–	1.55 a	–	–	0.53 a	–	–

*Values expressed as mean of three replications (n = 3) of each plant species. The Tukey's significance at P ≤ 0.05 among Se treatments is indicated by different letters within the plant species for each given nutrient.*

*SN, scallions; BL, basil; CO, cilantro; FW, fresh weight; Se, selenium; S, sulfur; Na, sodium; K, potassium; P, phosphorus; Ca, calcium; Mg, magnesium*.

Sodium accumulation demonstrated a trend (*P* = 0.0547) for the interaction of Se treatment and plant species. The addition of Na_2_SeO_4_ to the nutrient solution increased the Na content in scallions and cilantro (*P* ≤ 0.05) but not in basil (*P* = 0.0531) (Table 1). The potassium content increased by 91.6% in scallions that were treated with 10.0 mg/L Se, and it increased by 72.2% in basil that were treated with 5.0 mg/L Se (Table 1). Biofortification of cilantro did not impact the content of K or other minerals, except for Se and Na. The phosphorus content was significantly increased in scallions by 47.4% at 10.0 mg/L Se and increased in basil by 42.4% at 5.0 mg/L Se (Table 1). The contents of Ca and Mg were not affected by Se biofortification in basil or cilantro microgreens; however, both minerals increased in scallions at 10.0 mg/L Se by 59.8% for Ca and 60.6% for Mg (Table 1).

A significant increase of Cu in scallions started at the 5.0 mg/L Se treatment and continued to increase at 10.0 mg/L Se (Table 2). The contents of Fe, Mn, Zn, and B in scallions were highest (*P* ≤ 0.05) in the 10.0 mg/L Se treatment compared with the other Se doses. Basil and cilantro did not demonstrate significant changes in Cu, Fe, Mn, Zn, or B (Table 2). Overall, fresh Se-biofortified scallions demonstrated increases in the content of all other minerals that were analyzed, including major and trace minerals in human nutrition. Meanwhile, Se-biofortified basil and cilantro demonstrated increases in only three (S, P and K) and one (Na) minerals, respectively.

**Table 2:** Trace mineral content of scallion (*Allium fistulosum* L.), basil (*Ocimum basilicum* L.), and cilantro (*Coriandrum sativum* L.) microgreens treated with various concentrations of selenium as sodium selenate on a fresh weight basis.

**Treatment (mg·L^**−1**^ Se)**	**Cu**			**Fe**			**Mn**			**Zn**			**B**		
	**SN**	**BL**	**CO**	**SN**	**BL**	**CO**	**SN**	**BL**	**CO**	**SN**	**BL**	**CO**	**SN**	**BL**	**CO**
	**μg·g** ^****−1****^ **FW**														
0.0	0.25 c	0.34 a	0.48 a	6.71 b	8.90 a	6.73 a	2.78 b	3.71 a	4.40 a	1.57 b	1.49 a	3.52 a	2.23 b	3.78 a	5.44 a
2.5	0.28 c	0.39 a	0.46 a	5.64 b	7.29 a	11.44 a	3.26 b	4.43 a	3.46 a	1.69 b	1.68 a	3.39 a	2.70 b	4.29 a	4.85 a
5.0	0.35 b	0.41 a	0.62 a	8.00 b	7.30 a	10.44 a	3.30 b	5.00 a	4.10 a	1.88 b	1.94 a	4.20 a	2.77 b	3.96 a	5.54 a
10.0	0.48 a	–	–	14.62 a	–	–	5.65 a	–	–	3.10 a	–	–	5.40 a	–	

*Values expressed as mean of three replications (n = 3) of each herb species. The Tukey's significance at P ≤ 0.05 among selenium treatments is indicated by different letters within the plant species for each given nutrient.*

*SN, scallions; BL, basil; CO, cilantro; FW, fresh weight; Se, selenium; Cu, copper; Fe, iron; Mn, manganese, Zn, zinc; B, boron*.

### Total Phenolic Content and Total Antioxidant Capacity

**Figure 4:**
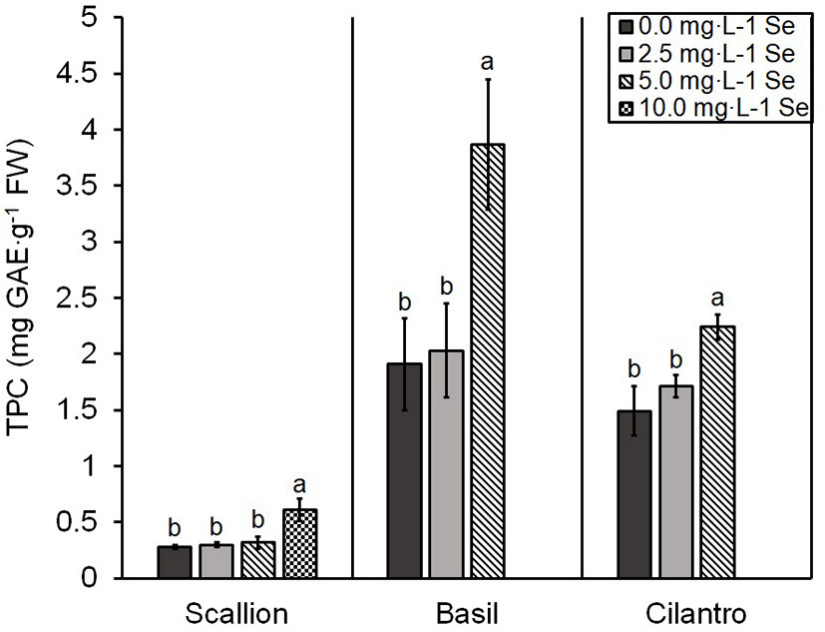
Total phenolic content (TPC) of scallion (*Allium fistulosum* L.), basil (*Ocimum basilicum* L.), and cilantro (*Coriandrum sativum* L.) microgreens treated with various concentrations of selenium (Se) as sodium selenate on a fresh weight (FW) basis. Bars indicate mean ± standard deviation (SD) for three replicates (*n* = 3) of each plant species. The Tukey's significance at *P* ≤ 0.05 among Se treatments is indicated by different letters within the plant species.

TPC was significantly higher at the treatment of highest Se concentration in all herb species (Figure 4). TPC demonstrated an interaction of Se treatment and plant species (*P* = 0.0001). For basil and cilantro, the highest dose of 5.0 mg/L Se treatment achieved the highest TPC (102.6 and 50.3% increases for basil and cilantro, respectively). For scallions, the TPC of 5.0 mg/L Se treatment was not affected; however, the highest dose of 10.0 mg/L Se treatment resulted in a 113.7% increase of TPC (Figure 4).

**Figure 5:**
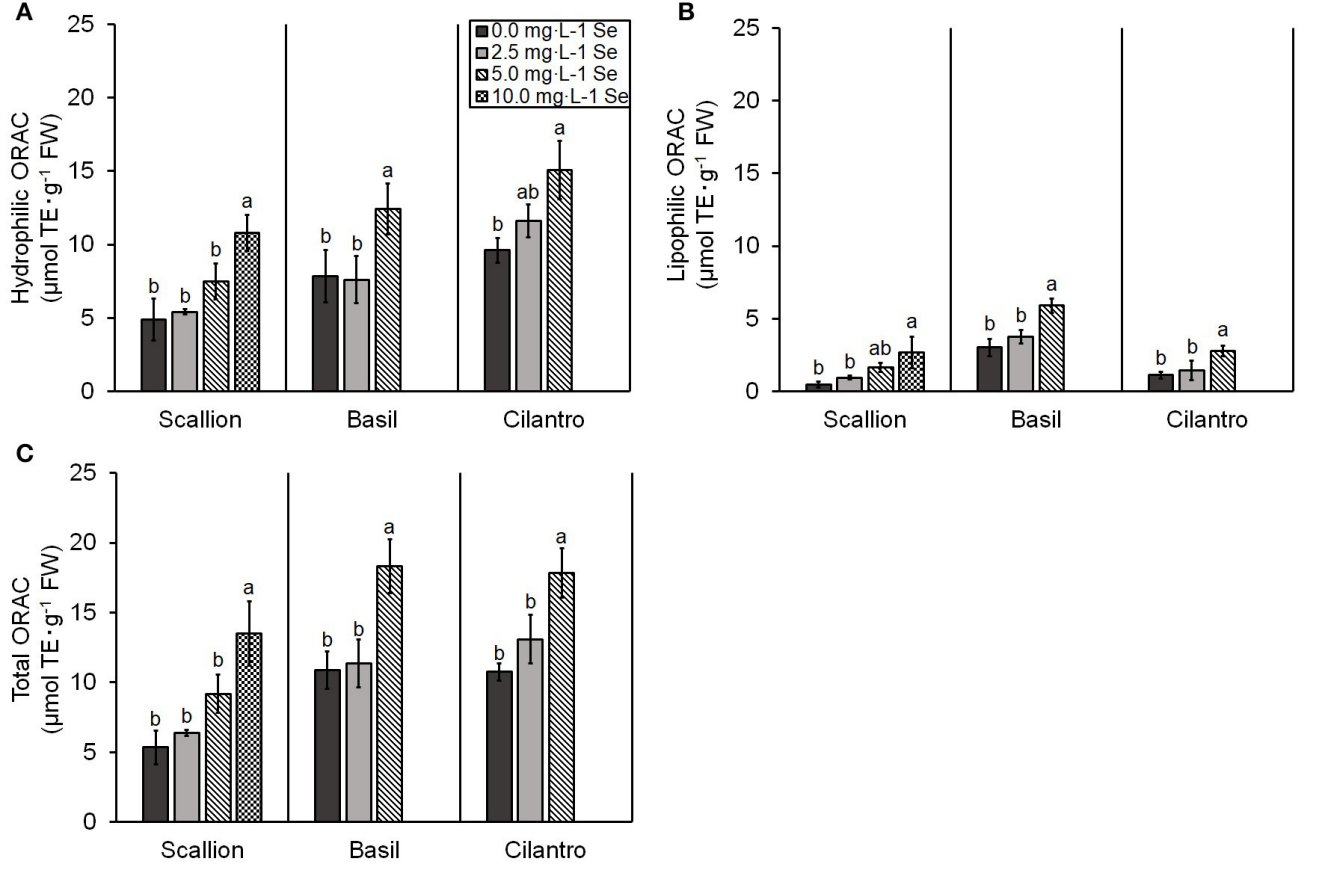
**(A)** Hydrophilic, **(B)** lipophilic, and **(C)** total oxygen radical absorbance capacity (ORAC) values for scallion (*Allium fistulosum* L.), basil (*Ocimum basilicum* L.), and cilantro (*Coriandrum sativum* L.) microgreens treated with various concentrations of selenium (Se) as sodium selenate on a fresh weight (FW) basis. Bars indicate mean ± standard deviation (SD) for three replicates (*n* = 3) of each plant species. The Tukey's significance at *P* ≤ 0.05 among Se treatments is indicated by different letters within the plant species.

In terms of total antioxidant capacity for all herb species, the hydrophilic, lipophilic, and total ORAC values were higher (*P* ≤ 0.05) at the highest Se treatment (Figures 5A–C). Only the lipophilic antioxidant capacity showed an interaction of Se treatment and plant species (*P* = 0.0227). Scallions at the highest dose of 10.0 mg/L Se and basil and cilantro at the highest dose of 5.0 mg/L Se resulted in herbs with the highest total antioxidant capacity determined by ORAC (152.2, 68.6, and 66.0% increases in scallions, basil, and cilantro, respectively) (Figure 5C).

## Discussion

The results confirm the role of scallion as a Se accumulator and indicate hyperaccumulator status as shown by the Se content in levels higher than 1,000 μg/g DW (Figure 1B) and superior accumulation capacity (Figure 2). It is also noteworthy that despite requiring the least amount of Se treatment volume (2,835 mL), scallions accumulated the highest content of Se at 2.5 and 5.0 mg/L Se treatments compared with basil and cilantro, which required 2,965 and 3,825 mL of treatments, respectively. Gupta and Gupta (2017) defined secondary accumulators as plant species that are capable of accumulating Se from 100 to 1,000 μg/g DW. Based on this definition, basil and cilantro can be classified into secondary Se accumulators, given the Se content demonstrated in this study (Figure 1B). The total Se uptake in basil plants was higher in the current study compared with the study by Puccinelli et al. (2019), which reported a maximum of 203 μg/g Se DW in basil microgreens grown from the seeds of Se-biofortified parent plants. Kopsell et al. (2009) assessed the Se content of a 32.0 mg/L Se foliar application to adult cilantro plants, yielding 9.3–49.5 μg/g Se DW in cilantro. These lower Se contents compared with those in our study support the efficiency of Se uptake under hydroponic conditions. Additionally, the high Se content of herbs in the current study may be a result of the addition of fresh nutrient solution every day, which provided a constant supply of Se to maximize the uptake in these young plants. Furthermore, the smaller biomass of microgreens may concentrate the Se content, as a “dilution effect” of minerals has been observed as basil plants mature and biomass increases (Puccinelli et al., 2017b). A study by Pannico et al. (2020), which used hydroponics in selenate biofortification of microgreens, demonstrated lower levels of Se content for green basil and cilantro (150.0 and 26.2 μg/g DW, respectively) than our study, likely due to the lower Se dose used (1.26 mg/L Se).

At high Se doses, growth stunting is a common symptom of Se toxicity in plants (Terry et al., 2000). In our study, the highest treatments of Se decreased the yield of both scallions and basil but did not affect cilantro (Figure 3). The similarity between Se and S can cause the substitution of S-amino acids with Se-amino acids in proteins and detrimental changes in the tertiary structure of the protein, resulting in Se toxicity in plants (Gupta and Gupta, 2017). The preferential uptake of selenate or sulfate varies among plant species (Terry et al., 2000), and in the present study, an increased S content was observed in scallions and basil but not in cilantro, which may account for the lack of effect on the yield of cilantro. A study on hydroponically grown lettuce shoots biofortified with selenate also demonstrated an increased S content at higher levels of Se application (Hawrylak-Nowak, 2013). This is significant for human nutrition since organic S can be used to increase synthesis of glutathione, the most abundant endogenous non-protein thiol, which has a protective role against free radical damage (Parcell, 2002).

The Dietary Guidelines for Americans (DGAs) recommend restricting Na consumption to <2,300 mg/day (U.S. Department of Agriculture and U.S. Department of Health Human Services, 2020), so the increased Na content of scallions and basil is not favorable. However, the highest amount of Na accumulation was 0.053 mg/g FW in scallions treated with 10.0 mg/L Se (Table 1). This translates to ~1.0 mg of Na for 20 g of fresh scallion microgreens; thus, the amount of Na from fresh scallion microgreens is negligible. Furthermore, the DGAs suggest the use of herbs and spices instead of salt as a strategy to lower Na intake (U.S. Department of Agriculture and U.S. Department of Health Human Services, 2020). An inadequate K intake can increase the risk for hypertension (Adrogué and Madias, 2014); thus, the increased K content of scallions and basil in this study is beneficial to human health. Previous studies on Se biofortification using microgreen basil and cilantro under hydroponic conditions have demonstrated an increase in K content on a DW basis compared with control plants (Pannico et al., 2020). Other studies using garlic reported a decrease in K content, while biofortification of onion did not demonstrate significant K changes (Põldma et al., 2011, 2013). However, the studies on garlic and onion involved mature plants instead of microgreens. The concentration of K is typically higher in young plant tissues because of the role of K in photosynthesis, respiration, and water homeostasis (Waterland et al., 2017).

Other major minerals of importance in human health are P, Ca, and Mg, which are essential components for bone formation and strength, with deficiencies contributing to the risk for osteoporosis (Bonjour et al., 2009). While P deficiencies in humans are rare due to its ubiquity in food, inadequate Ca intake may result from diets excluding dairy products, and Mg intake is often suboptimal due to its removal during processing of staple foods (Bonjour et al., 2009). Pannico et al. (2020) demonstrated increased P on a DW basis for green basil and cilantro microgreens biofortified with 0.63 and 1.26 mg/L Se as selenate. For cilantro, the lack of effect on P in our study may be a result of the different dosages used for this species between studies. Põldma et al. (2013) further reported a decreased Ca content in onions grown with a foliar spray of 10 and 50 mg/L Se as selenate and an increased Mg content at 50 mg/L Se. In the present study, the increases of P, Ca, and Mg in scallions are beneficial to human health. Overall, biofortification of fresh scallions resulted in enhancements of major minerals in human nutrition involved in fluid balance and bone health.

Trace elements also contribute to bone mineralization, but Cu, Fe, Mn, and Zn are more often studied as cofactors for antioxidant enzymes, such as superoxide dismutase and catalase (Soetan et al., 2010). Micronutrient deficiencies are a major public health concern, with Fe deficiency of specific interest, especially among women and children (Soetan et al., 2010). The enhanced content of Cu, Fe, Mn, and Zn demonstrated in scallion microgreens treated with 10.0 mg/L Se adds to the nutritional benefits of Se-biofortified scallions. Other Se biofortification studies that analyzed trace mineral contents have variable results due to differences in plant species, Se species, application technique, and dosage (He et al., 2004; Boldrin et al., 2013; Pannico et al., 2020). The increased content of B in scallions at 10.0 mg/L Se observed in the present study is also favorable for humans and plants. Boron has several roles in human health including steroid hormone metabolism, bone development, and cell membrane maintenance (Uluisik et al., 2018). In plant nutrition, B is essential for plant growth and phenolic metabolism (Uluisik et al., 2018). This is important since plants are a major source of phenolic compounds in the human diet.

A diet rich in phytochemicals is associated with lower incidence of chronic disease (Zhan et al., 2018). The accumulation of polyphenols in plants increases under stressful growing conditions, such as in the presence of excess Se in the soil (Kulbat, 2016; Saha et al., 2017). The increased TPC in Se-biofortified scallion, basil, and cilantro microgreens in the current study parallels the results of earlier studies in adult basil and onion and microgreen green basil and cilantro (Hawrylak-Nowak, 2008; Põldma et al., 2013; Pannico et al., 2020). Zheng and Wang (2001) reported the TPC of 2.23 mg GAE/g FW for basil, and Zhan et al. (2018) reported 0.20 mg GAE/g FW for scallions. These values are comparable with the control groups in our study (1.91 and 0.29 mg GAE/g FW for control basil and scallions, respectively). Henning et al. (2011) demonstrated the TPC of basil and cilantro at 8.70 and 6.10 mg GAE/g FW, respectively. In comparison, the TPC values were lower in our study for control basil and cilantro at 1.91 and 1.49 mg GAE/g FW, respectively. These lower values may be due to the difference in plant maturity. For instance, McCance et al. (2016) reported a decreased TPC in purple basil at earlier stages of harvest. TPC can also vary depending on the growing conditions and extraction techniques. Enhanced TPC adds to the nutritional benefits of biofortification with 5.0 mg/L Se in basil and cilantro and 10.0 mg/L Se in scallions. The TPC in scallions at 5.0 mg/L Se was not affected, which may be a result of the increased capacity of *Alliums* to resist Se toxicity.

The total antioxidant capacity of foods can be assessed by ORAC and provides a unique *in vitro* assessment of hydrophilic and lipophilic antioxidants, which considers the most common free radical in lipid oxidation *in vivo* (Shahidi and Zhong, 2015). Similar to the results in our study, Guardado-Felix et al. (2017) demonstrated an increase in ORAC for chickpea sprouts treated with increasing Se levels. In the current study, the highest Se treatment increased ORAC but decreased plant yield in scallions and basil. At high treatment doses, Se can act as a prooxidant in plants causing oxidative stress, which contributes to Se toxicity (Saha et al., 2017). To counteract the oxidative stress, plants increase antioxidant defenses (Gupta and Gupta, 2017). However, toxicity symptoms, such as decreased growth, can result if reactive oxygen species (ROS) overwhelms these antioxidant defenses. This may explain the increase in total ORAC occurring alongside a decreased yield for basil and scallions in our study. However, cilantro demonstrated increases in ORAC without decreased plant yield, suggesting antioxidant defenses of cilantro were adequate to quench ROS induced by Se.

## Conclusion

Sodium selenate biofortification affected the content of dietary minerals, TPC, and total antioxidant capacity in the culinary herb microgreens studied. The Se content, TPC, and total antioxidant capacity increased at the highest Se dose for all herb species. Scallions accumulated the highest total Se, suggesting that scallion microgreens have the greatest potential as a functional food despite decreased crop yield at its highest dose. In populations with adequate Se consumption, increasing Se intake using Se-rich dietary sources may be more beneficial than over-the-counter supplements which risk toxicity. A realistic serving size of 10 fresh scallion microgreens treated with 10.0 mg/L Se would supplement about 32 μg of Se to the diet. While scallions at 10.0 mg/L Se treatment may be unfavorable from the perspective of a grower due to the decreased crop yield, scallions offer a dietary supplement of Se with added benefits of increased TPC, antioxidant capacity, and content of other minerals important to human health. At the lower 5.0 mg/L Se treatment, scallion yield was not affected; however, other minerals and antioxidants were not increased. Basil and cilantro at 5.0 mg/L Se provided less Se but increased antioxidants. Se-biofortified basil offers the additional benefit of elevated S, K, and P contents, while the absence of decreased crop yield in cilantro can make cilantro preferable to growers. A serving size of 10 fresh microgreens of basil or cilantro at 5.0 mg/L Se would be approximately 19 and 15 μg of Se, respectively. These Se-biofortified herb microgreens can be added to meals for flavor while providing a simple method of enhancing dietary Se intake. Different Se dosages and application techniques impact plant yield and mineral and antioxidant contents of biofortified foods, requiring additional studies that consider a variety of biofortification techniques to generate optimal growing recommendations. The results of this study confirm that sodium selenate biofortification of culinary herb of culinary herb microgreens under hydroponic conditions can produce functional foods by increasing the Se content with additional benefits of enhanced dietary minerals and antioxidants.

## Data Availability Statement

The original contributions generated for the study are included in the article/Supplementary Material, further inquiries can be directed to the corresponding author/s.

## Author Contributions

RN, YM, JT, and NW conceived the project. RN carried out experiment, performed analyses, and wrote the original draft manuscript. CS performed the analysis of mineral contents. RN, YM, CS, JT, and NW assisted with study design and experiments. All authors read and contributed to earlier versions and approved the final version.

## Conflict of Interest

The authors declare that the research was conducted in the absence of any commercial or financial relationships that could be construed as a potential conflict of interest.

## Publisher's Note

All claims expressed in this article are solely those of the authors and do not necessarily represent those of their affiliated organizations, or those of the publisher, the editors and the reviewers. Any product that may be evaluated in this article, or claim that may be made by its manufacturer, is not guaranteed or endorsed by the publisher.
